# First report of *Meloidogyne javanica* infecting *Zinnia elegans* in Ceará State, Brazil

**DOI:** 10.21307/jofnem-2020-066

**Published:** 2020-07-28

**Authors:** Francisco Jorge Carlos Souza Junior, Mayara Castro Assunção

**Affiliations:** Departamento de Agronomia, Universidade Federal Rural de Pernambuco, Dois Irmãos 52.171-900, Recife, PE, Brazil

**Keywords:** *Meloidogyne javanica*, Molecular biology, Morphology, Ribosome markers, Root-knot nematodes, *Zinnia elegans*

## Abstract

Roots of *Zinnia elegans* exhibiting galls were observed and collected in the city of Pacoti, CE, Brazil. Through the morphological characterization and molecular analysis of the ITS and 28S rDNA regions, the causal agent was identified as a nematode of the genus *Meloidogyne* species *M. javanica*. This is the first detection of *M. javanica* in *Zinnia elegans* in the state of Ceará in Brazil.

*Zinnia* (*Zinnia elegans* Jacq.), a herbaceous plant of the Asteraceae family, is among the species use in Brazil for flower production. This species, originally from Mexico, has a broad diversity of flower colors, a wide variety of petal shapes, and the possibility of being cultivated throughout the year (Torres, 1963; Stimart, 1987).

The *Zinnia* genus comprises 17 species and in Brazil it is known by common names as captain, young, and old, cinnamon sticks or *zinnia*, being grown in regions of mild temperature and in tropical areas. The plant has an erect shape, oval leaves, and colorful floral rays of various forms (Torres, 1963; Ahmad et al., 2015).

The genus *Meloidogyne* (Goeldi, 1887), also known as gall nematode, is widespread in all regions of Brazil, associated with agricultural crops. Alternative hosts, such as ornamental plants, maintain the inoculum and increase its populations, compromising the development of plants and affecting the quality of products and resulting in losses in productivity (Moens et al., 2009).

In October 2019, samples of roots of *Zinnia elegans* with galls caused by root-knot nematode were sampled in vegetable gardens in the city of Pacoti (4° 13′ 45″S; 38° 55′ 26″ W), in the state of Ceará, Brazil. Nematodes in the roots were processed for extraction using the protocol given by Coolen and D’Herde (1972). For the morphological characterization of the species, 20 females and juveniles of second stage were measured and perineal pattern from 20 females were prepared according to Taylor and Netscher (1974). The determination of the esterase profile was made according to Carneiro and Almeida (2001), using 20 female.

For molecular identification, the D2 to D3 region of 28S rDNA segment was amplified and sequenced using the primers D2A (5′-ACAAGTACCGTGAGGGAAAGTTG-3′) and D3B (5′-TCGGAAGGAACCAGCTACTA-3′) (De Ley et al., 1999) and ITS primers with VRAIN2F (5′-CTTTGTACACACCGCCCGTCGCT-3′) and VRAIN2R (5′-TTTCACTCGCCGTTACTAAGGGAATC-3′) (Vrain et al., 1992).

The consensus sequences were assembled from the forward and reverse sequences, using the Staden package (Staden et al., 2000). All consensus sequences obtained were used to compare with the NCBI nucleotide database, based on the research using the BLAST algorithm. Several sequence alignments for each individual gene were generated with the online version of MAFFT version 7 with the iterative refinement method L-INS-i (Katoh and Toh, 2008; Katoh, 2013). Phylogenetic analysis was performed using the maximum likelihood (ML) methods for individual genes, performed via RAxML-HCP2 v.8.2.8 (Stamatakis, 2014) implemented in CIPRES Portal v.2.0 (https://www.phylo.org/portal2/home.action) with 1,000 repetitions. For the ML analysis, a GTR-GAMMA model of evolution was used.

In females (*n* = 20), the body length was 437.3 ± 34.1 (427.1-514.3) μm; the stylus measured 12.4 ± 0.4 (11.4-14.1) μm in length; the DGO was 3.0 ± 0.6 (2.8-4.5) μm. For second-stage juveniles (*n* = 20), the body length was 334.0 ± 24.3 (327.0-417.8) μm; stylus length 11.0 ± 1.4 (10.2-12.0) μm; DGO equal to 2.6 ± 0.5 (2.0-2.8) μm; and tail measurements *c* = 9.1 ± 0.8 (7.4-10.2) μm and *c*′ = 5.0 ± 0.6 (4,7-5.7) μm, showing tapering in the terminal region (Mai and Mullin, 1996). The perineal patterns of the females were rounded with the low dorsal arch, smooth streaks, and parallel lateral lines highlighted on both sides (Fig. 1A). The polymorphisms of the esterase bands by electrophoresis revealed the phenotype J3 (*Rm* = 1.00, 1.25, and 1.40) typical of *M. javanica* (Fig. 1B).

**Figure 1:**
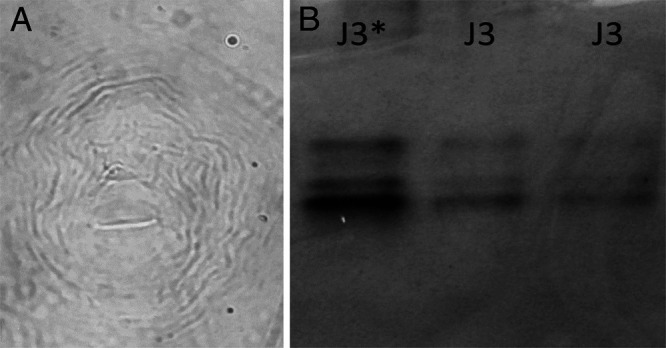
(A) Perineal pattern of *Meloidogyne javanica,* (B) Esterase phenotypes of *M. javanica* detected in *Zinnia elegans* (J3: *M. javanica*; J3*: *M. javanica* reference isolate).

The sequences submitted to GenBank ITS: MT337435 and D2 to D3 28S: MT341299 of the studied rDNA regions, showed 98 to 99% identity with sequences of *M. javanica* isolates from Brazil, China, and India. Phylogenetic analyzes of these sequences, using ML, placed the *Meloidogyne* population (CN0027) isolated from *Zinnia elegans* in a clade with the *M. javanica* sequences from GenBank, confirming it as *M. javanica* (Fig. 2).

**Figure 2:**
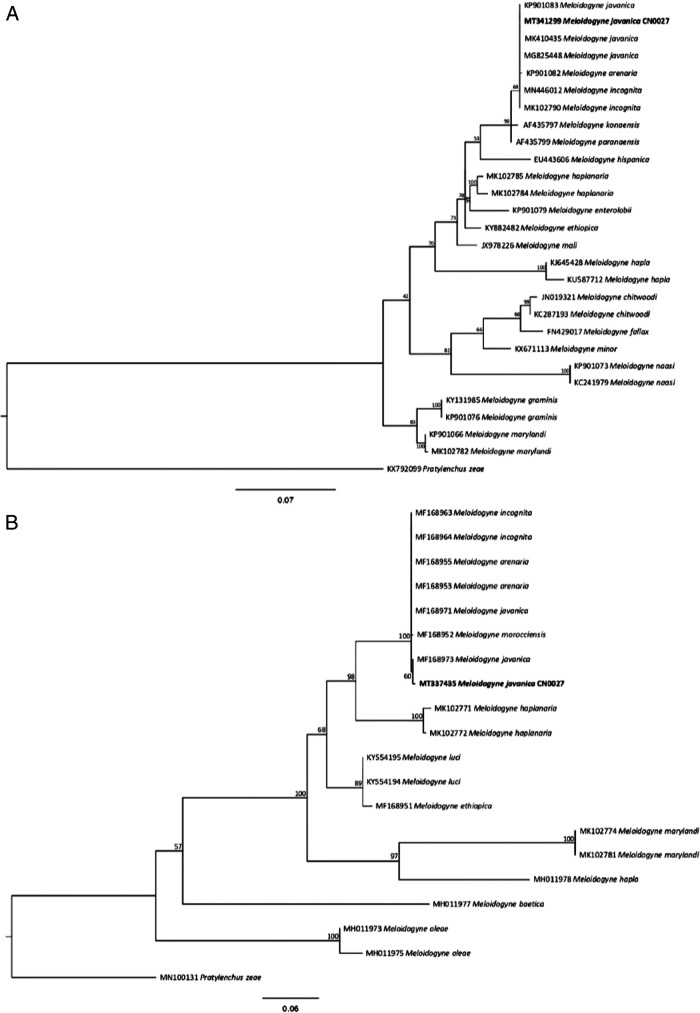
Phylogenetic relationship of *Meloidogyne javanica* from Ceará, Brazil, based on alignment of the sequence of the (A) D2/D3 expansion fragments of 28S rDNA and (B) sequence of ITS. The phylogenetic tree was estimated by maximum likelihood. *Pratylenchus scribneri* and *P. zeae* was used an outgroup.

In 2014, *M. javanica* was reported associated with *Z. elegans* in Iraq (Ali et al., 2014) and, in Brazil in 2017, the species *M. incognita* (Moreira et al., 2017) and *M. hapla* (Freire and Mosca, 2009) were described in *Z. elegans*. This nematode has economic importance in the production areas, with wide geographical distribution and occupying the first position in the world ranking among the nematodes harmful to agricultural crops (Jones et al., 2013)

However, this is the first record of *M. javanica* parasitizing roots of *Z. elegans* in Brazil, and thus, this work updates the information on the occurrence of this species in *Z. elegans*, indicating this plant as a potential host for this nematode in the state of Ceará.
